# Melittin derived peptide-drug conjugate, M-DM1, inhibits tumor progression and induces effector cell infiltration in melanoma by targeting M2 tumor-associated macrophages

**DOI:** 10.3389/fimmu.2023.1178776

**Published:** 2023-04-14

**Authors:** Chanmi Jeong, Jeongdong Kim, Ik-Hwan Han, Soyoung Kim, Ilseob Choi, Hongsung Kim, Jin-Hyun Jeong, Hyunsu Bae

**Affiliations:** ^1^ Department of Physiology, College of Korean Medicine, Kyung Hee University, Seoul, Republic of Korea; ^2^ Twinpig Biolab Inc. Research & Development Center, Seoul, Republic of Korea; ^3^ College of Pharmacy, Yonsei Institute of Pharmaceutical Sciences, Yonsei University, Incheon, Republic of Korea; ^4^ Convergence Innovation Support Center, Gangwon Technopark, Chuncheon-si, Gangwon-do, Republic of Korea; ^5^ Department of Korean Medicine, College of Korean Medicine, Kyung Hee University, Seoul, Republic of Korea

**Keywords:** peptide-drug conjugate, melittin, mertansine, tumor associated macrophages, melanoma

## Abstract

**Background:**

Melanoma has the highest mortality rate among all the types of skin cancer. In melanoma, M2-like tumor-associated macrophages (TAMs) are associated with the invasiveness of tumor cells and a poor prognosis. Hence, the depletion or reduction of M2-TAMs is a therapeutic strategy for the inhibition of tumor progression. The aim of this study was to evaluate the therapeutic effects of M-DM1, which is a conjugation of melittin (M), as a carrier for M2-like TAMs, and mertansine (DM1), as a payload to induce apoptosis of TAMs, in a mouse model of melanoma.

**Methods:**

Melittin and DM1 were conjugated and examined for the characterization of M-DM1 by high-performance liquid chromatography and electrospray ionization mass spectrometry. Synthesized M-DM1 were examined for *in vitro* cytotoxic effects. For the *in vivo* study, we engrafted murine B16-F10 into right flank of C57BL/6 female mice and administered an array of treatments (PBS, M, DM1, or M-DM1 (20 nmol/kg)). Subsequently, the tumor growth and survival rates were analyzed, as well as examining the phenotypes of tumor-infiltrating leukocytes and expression profiles.

**Results:**

M-DM1 was found to specifically reduce M2-like TAMs in melanoma, which potentially leads to the suppression of tumor growth, migration, and invasion. In addition, we also found that M-DM1 improved the survival rates in a mouse model of melanoma compared to M or DM1 treatment alone. Flow cytometric analysis revealed that M-DM1 enhanced the infiltration of CD8+ cytotoxic T cells and natural killer cells (NK cells) in the tumor microenvironment.

**Conclusion:**

Taken together, our findings highlight that M-DM1 is a prospective agent with enhanced anti-tumor effects.

## 1 Introduction

Melanoma that originates from melanocytes is most aggressive and life-threatening malignancy in skin cancer, which includes basal cell carcinoma (BCC) and squamous cell carcinoma (SCC). Its incidence has been rising rapidly over the last 50 years (1, 2). Melanoma accounts for about 1% of all skin cancers, but causes the majority of skin cancer-related deaths because of its ability to rapidly spread to other organs (3, 4). However, the incidence rate is higher in women than in men, especially in individuals under the age of 30 (5). In early-stage malignant melanoma without regional lymph node infiltration, surgical resection is usually an effective treatment. However, the 5-year survival rate is poor after the melanoma has metastasized to lymph nodes or other organs (6, 7). Recently, immunotherapy drugs, such as cytokines and immune checkpoint inhibitors, have emerged for use in melanoma patients, with relevant treatments including the use of immunotherapy drugs alone or in combination (8). The tumor microenvironment (TME) of melanoma and the presence of tumor-associated macrophages (TAMs) have been shown to be related to tumor growth, invasion, metastasis, and drug resistance. The molecular mechanisms leading to its onset and progression are the focus of intensive investigation with the aim of developing novel therapeutic strategies (9, 10).

Macrophages are polarized in two main directions: classically activated pro-inflammatory M1 macrophages, which have anti-tumor properties, and activated anti-inflammatory M2 macrophages, which promote tumor growth and are immunosuppressive (11–13). M2-like TAMs are the main cells that produce cytokines, chemokines, and growth factors and induce the secretion of inhibitory immune checkpoint proteins from T cells in TME (14, 15). An abundance of M2-like TAMs in the TME is associated with immunosuppression during tumor growth and a poor prognosis (16). TAMs play a major role in diverse aspects of tumor progression (17–19). Because of the pivotal role of TAMs in supporting tumor progression, they have emerged as an attractive target for cancer therapy (20–23).

Recently, peptides that offer versatility in drug discovery for the successful treatment of cancers have emerged. Peptide-drug conjugates (PDCs) represent an important therapeutic strategy for increasing tumor penetration and selectivity (24–26). We previously reported that M, which is extracted from bee venom, targets M2 macrophages and improves tumor treatment in lung cancer (27). M is an amphipathic peptide with 26 amino acid residues (AIGAVLKVLTTGLPALISWIKRKRQQ) that specifically binds to M2-like TAMs (28). Mertansine (DM1) is a strong cytotoxic agent that interacts with tubulin and inhibits the assembly of tubulin into microtubules. Because it targets microtubules and inhibits cell cycle, its clinical efficacy as a potential anti-cancer agent has been studied (29). However, meaningful results have yet to be obtained in clinical trials, with patients also suffering several side effects, such as myelosuppression (30, 31). Antibody drug conjugates (ADCs) are currently the most successful type of drug conjugates, consisting of antibody (targeting), linker (linking the antibody to the payload), and Payload (killing target cells). Payload is a cytotoxic compound and is divided into microtubule inhibitors, such as DM1, and DNA-damaging agents, such as anthracyclines (32). DM1 alone has not been developed as a drug but is currently used as an antibody-drug conjugate (33, 34). It has been reported that the peptide LLC2B combined with DM1 exerts an anticancer effect in breast and esophageal squamous cell carcinoma, suggesting it could be developed as a potential PDC for tumor treatment (35). In the present study, we investigated the anti-cancer effects of M-DM1, which was synthesized using M as a carrier for targeting TAMs and DM1 as a payload. We found that M-DM1 exerts its therapeutic effects by specifically depleting M2-like TAMs in a melanoma mouse model. Through the regulation of M2-like TAMs, M-DM1 induced a significant increase in the infiltration of effector cells, such as CD8 T cells and natural killer (NK) cells, into the TME. Collectively, our results suggest that depleting M2-like TAMs in the TME by treatment with M-DM1 has immunotherapeutic effects in malignant melanoma.

## 2 Materials and methods

### 2.1 Conjugation and characterization of M-DM1 conjugates

Maleimide-modified melittin was purchased from GenScript (Beijing, China). DM1 was purchased from MedChem Express (Princeton, NJ, USA). Dimethylformamide (DMF), boric acid, and ultrafilter (3 kDa MWCO) were purchased from Sigma-Aldrich (St. Louis, MO, USA). Distilled water, acetonitrile, and formic acid were purchased from Thermo Fisher Scientific (Waltham, MA, USA) and used in the LC-MS analysis.

With regards to the conjugation of DM1 to M, DM1 was coupled to the N-term of maleimide-modified M. The resulting PDC was denoted as M-DM1. M (100 µM, 1 mL, 0.1 mmoL) in 25 mM sodium borate buffer (25 mM NaCl, 1 mM EDTA, pH 8.0) was added to DM1 (1.1 eq., 10 mM in DMF) and DMF (10% v/v). The reaction was incubated at 37 °C for 2 h under mild agitation. The crude product was filtered three times by ultrafiltration in Dulbecco’s phosphate buffered saline (DPBS) (Welgene, Republic of Korea). M-DM1 was characterized using reverse-phase high-performance liquid chromatography (HPLC) on a Poroshell 120 C18 column (2.7 µm, 3 × 50 mm, Agilent, Santa Clara, CA, USA) with an injection volume of 5 µL and a flow rate of 0.5 mL/min. Mobile phases A and B were prepared using 0.1% formic acid in HPLC grade water and acetonitrile. The solvent gradient on the Agilent HPLC Infinity 1260 system was programmed from 5% B to 95% B in 55 min after a 5- min isocratic hold at 5% B. Identification of the M-DM1 was confirmed by Agilent 6530 Q-Tof with electrospray ionization.

### 2.2 Cell culture

The murine melanoma cell line B16F10 (Korean Cell Line Bank (KCLB)) was cultured in Dulbecco’s modified Eagle’s medium (DMEM) (Welgene, Gyeongsan, South Korea) supplemented with heat-inactivated fetal bovine serum (FBS) (Welgene) and 1% penicillin/streptomycin (Hyclone, Logan, UT, USA). Human melanoma cell line SK-MEL-5 (KCLB) was cultured in Minimum Essential Medium (MEM) (Welgene), and human monocytic leukemia cell line THP-1 (KCLB) was cultured in RPMI 1640 medium (Welgene) supplemented with 10% inactivated FBS and 1% penicillin/streptomycin (Hyclone). The cells were cultured every 2–3 days until the cells became 80% confluent, after which these were incubated at 37°C with 95% humidity and 5% CO_2_ for all experiments.

### 2.3 *In vitro* macrophage differentiation and conditioned medium preparation

THP-1 monocytes were differentiated into macrophages by treatment with 100 nM phorbol-12-myristate-13-acetate (PMA) (Sigma-Aldrich) for 24 h. The cells were treated with RPMI1640 supplemented with 10% FBS for 48 h to obtain differentiated M0 macrophages. THP-1 cells were polarized into M1 macrophages by treatment with 100 ng/mL lipopolysaccharide (Sigma-Aldrich) and 20 ng/mL recombinant human interferon-γ (rhIFN-γ) (Peprotech, Rochy Hill, NJ, USA). To obtain M2 macrophages, cells were incubated with 20 ng/mL recombinant human interleukin (rhIL)-4 (Peprotech) and 20 ng/mL rhIL-13 (Peprotech). To prepare tumor-conditioned medium, SK-MEL-5 cells were cultured in serum-free medium for 24 h. The supernatant was then collected and filtered using 0.2-μM syringe filters (GVSm Sanford, ME, USA). To induce differentiation into the tumor-associated macrophages (TAMs), THP-1 cells treated with PMA were incubated with 20% of the tumor-conditioned medium.

### 2.4 Transwell assay

The invasive and migratory ability of melanoma cells following treatment with TAM supernatant was examined using a transwell chamber (8.0 μm pore; SPL Life Sciences, Pocheon, South Korea). For the invasion assay, the transwell membrane was pre-coated with Matrigel diluted 1:9 with serum-free MEM for 24 h at 37 °C. In the upper chamber, SK-MEL-5 cells were seeded at a density of 5 × 10^4^ cells/well, and in the bottom chamber, 2 μM of M-DM1, DM1, or M were added along with TAM supernatant. After 24 h of incubation, the upper chamber was washed with PBS and was fixed with 4% paraformaldehyde. The upper chamber was then stained using 1% crystal violet for 20 min. The migration assay was carried out, and the number of migrating cells was measured according to the aforementioned method, without pre-coating with Matrigel. Five or more different fields were photographed for the invasion or migration site of each sample using an inverted microscope (Olympus, Tokyo, Japan). The number of cells was measured using ImageJ (National Institutes of Health (NIH), Bethesda, MD, USA.

### 2.5 Colony formation assay

To investigate the inhibition of cell proliferation with or without peptides, a colony formation assay was performed using a transwell chamber (8.0 μm pore; SPL Life Sciences). SK-MEL-5 cells (1 × 10^5^ cells/well) were seeded in the bottom chamber, and 2 μM of peptides were added along with the TAM supernatant in the upper chamber for 24 h. The cells were then washed and fixed using 4% paraformaldehyde for 15 min and stained with 1% crystal violet for 20 min. Five or more fields were photographed using an inverted microscope (Olympus). The number of cells was measured using ImageJ (NIH).

### 2.6 MTS assay

Cell viability was determined using an MTS assay kit (CellTiter 96^®^ Aqueous One Solution Cell Proliferation Assay; Promega, Madison, WI, USA) according to the manufacturer’s instructions. THP-1 cells (3 × 10^4^ cells/well) were seeded in a 96-well plate and polarized into M0, M1, M2 macrophages, or TAMs. TAMs were utilized along with 20% of tumor-conditioned medium. Following incubation 24 h at 37 °C, peptides (10, 5, 2.5, 1.25, 0.612, 0.3, and 0.15 μM) were added into each well along with a serum-free medium and incubated for 24 h. To assess the proliferation of melanoma cells, SK-MEL-5 cells were seeded at a density of 0.5 × 10^4^ cells/well in a 96- well plate. Cell death and proliferation were analyzed using an MTS assay. The cells were incubated with MTS reagent (Promega) diluted 1:5 in MEM serum-free media for 2 h. The absorbance was measured at 490 nm using a microplate reader (Molecular Devices, San Jose, CA, USA).

### 2.7 Tumor inoculation and *in vivo* study

C57BL/6 female wild-type mice (6–8 weeks old) were purchased from Jackson Laboratory (Bar Harbor, ME, USA). For the subcutaneous tumor model, B16-F10 cells were mixed with Matrigel matrix (Corning, NY, USA) and inoculated subcutaneously into the right flank (2 × 10^5^ cells/mouse) of the mice. PBS, M, DM1, or M-DM1 (20 nmol/kg) was injected intraperitoneally every 3 days five times, beginning on day 7 after tumor inoculation. All tumor tissues were harvested 21 days after tumor inoculation. Tumor size was examined with a digital caliper every 3 days, and tumor volume was calculated using the following formula: V = (width (2) × length)/2. Mice were sacrificed when the tumor size attained a maximum diameter of 1–1.5 cm after inoculation. The animal studies were approved by the Institutional Animal Care and Use Committee of Kyung Hee University (KHUASP(SE)-20–530).

### 2.8 Immunohistochemistry analysis

THP-1 cells were seeded at a density of 5 × 10^4^ cells/well on cover glass (Thermo Fisher Scientific, Waltham, MA, USA) in a 24- well plate. The cells were polarized into M0, M1, M2 macrophages, and TAMs using the aforementioned method and then incubated with M-DM1 (2 μM) for 24 h. The cells were washed with PBS, fixed with 4% paraformaldehyde for 15 min at room temperature, and blocked with 5% bovine serum albumin (BSA) in PBS for 1 h. The cover glasses were then incubated with anti-cleaved-caspase-3 antibody (1:400) (rabbit polyclonal; Cell Signaling Technology, Danvers, MA, USA) and 4’,6-diamidino-2-phenylindole (DAPI) to visualize the nuclei. All images were captured using a ZEISS LSM 800 laser scanning microscope (Bio-Rad, Richmond, CA, USA).

Immediately after mice sacrifice, the tumor tissues were harvested and fixed overnight in 4% paraformaldehyde before embedding in paraffin wax. Sections (4- μm- thick) were cut using a rotary microtome. The section slides were dipped in xylene and then 100%, 90%, 80%, and 70% ethanol solutions, respectively, before washing in running tap water for rehydration.

For immunofluorescence, the tumor tissue sections were heated in 10 mM sodium citrate buffer (pH 6.0) in an autoclave and further incubated for 15 min with 3% hydrogen peroxide (Sigma-Aldrich). After washing with PBS, nonspecific binding was reduced by blocking with 5% BSA in PBST (PBS with 0.2% Triton X-100) for 1 h. The slides were then incubated with anti-mouse CD163 antibodies (1:200; Abcam, Cambridge, UK) overnight at 4 °C, and then visualized with Alexa-594 conjugated anti-mouse secondary antibodies (1:400; Invitrogen, Carlsbad, CA, USA) for 2 h at 4 °C. Each slide was mounted in VECTASHIELD Mounting Medium with DAPI (Vector Laboratories, Burlingame, CA, USA) to visualize the nuclei and imaged with laser scanning confocal microscopy (Carl Zeiss, Jena, Germany).

The slides were incubated with PCNA rabbit polyclonal antibody (1:200, Abcam) diluted with 5% BSA in PBST (PBS with 0.3% Triton X-100) overnight at 4 °C. The sections were washed with PBS, incubated with a biotinylated secondary antibody, and visualized using DAB substrate kit (Vector Laboratories). The sections were then counterstained with hematoxylin and examined in at least three randomly selected fields. All images were captured using an LSM5 PASCAL (Carl Zeiss, Jena, Germany) and the total intensity was analyzed using the ImageJ (NIH).

### 2.9 Western blot analysis

THP-1 cells differentiated into M0, M1, M2 macrophages, and TAMs using the methods described above were incubat ed with M-DM1 (2 μM) for 24 h. The cells were washed and proteins were extracted using RIPA buffer (Thermo Fisher Scientific) supplemented with 0.1% sodium dodecyl sulfate (SDS) and protease inhibitor. The protein concentrations were measured using the BCA reagent kit (Thermo Fisher Scientific). Proteins were separated by 10% SDS–polyacrylamide gel electrophoresis and transferred to polyvinylidene difluoride membranes.

Total protein was extracted from tumor tissues using RIPA buffer and quantified with BCA assay. β-actin mouse polyclonal antibody (sc-1836, 1:2000; Santa Cruz, Dallas, TX, USA), PCNA mouse polyclonal antibody (sc-56, 1:1000; Santa Cruz), caspase-3 Antibody (#9662, 1:1000; Cell Signaling Technology, Danvers, MA, USA), and cleaved-caspase-3 (Asp175) (#9661, 1:1000; Cell Signaling Technology) were used as primary antibodies. Mouse anti-goat IgG-HRP (sc-2354, 1:2000; Santa Cruz), goat anti-rabbit IgG/HRP (SE134, 1:2000; Solarbio), and goat anti-mouse IgG/HRP (SE131, 1:2000; Solarbio) were used as secondary antibodies. The protein bands were detected using a chemiluminescence reagent kit (SurModics, Eden Prairie, MN, USA). The intensity of the protein was normalized to the intensity of β-actin using ImageJ (NIH).

### 2.10 Real-time quantitative PCR

Total RNA was isolated from tumor tissues using an easy-BLUE RNA extraction kit (iNtRON Biotechnology, Seongnam, South Korea). cDNA was synthesized using Cyclescript reverse transcriptase (Bioneer, Daejeon, South Korea) according to the manufacturer’s instructions. Quantitative real-time PCR was performed using SensiFAST SYBR no-Rox kit (Bioline, South Korea). The data were normalized to GAPDH levels. Each reaction was performed in triplicate. The primers used are listed in **Table 1**.

**Table 1:** Primers used in Real-Time PCR.

Gene	F/R	Primer Sequence
**GAPDH**	F	5’-ACC CAG AAG ACT GTG GAT GG-3’
	R	5’-CAC ATT GGG GGT AGG AAC AC -3’
**Arginase-1**	F	5’- AGA CAG CAG AGG AGG TGA AGA G-3’
	R	5’-CGA AGC AAG CCA AGG TTA AAG C-3’
**CD206**	F	5’-TCT TTG CCT TTC CCA GTC TCC-3’
	R	5’-TGA CAC CCA GCG GAA TTT C-3’
**IL-1β**	F	5’-GCC CAT CCT CTG TGA CTC AT-3’
	R	5’ AGG CCA CAG GTA TTT TGT CG-3’
**IL-10**	F	5’-CCA AGC CTT ATC GGA AAT GA-3’
	R	5’-TTT TCA CAG GGG AGA AAT CG-3’
**MMP9**	F	5’-TGA ATC AGC TGG CTT TTG TG-3’
	R	5’-ATT TTC CAG TAG GGG CAA CT-3’
**VEGF**	F	5’-CCA CGA CAG AAG GAG AGC AGA AGT CC-3’
	R	5’-AGG TAA CGC CAG GAA TTG TTG C-3’
**Snail**	F	5’-AAA CCC ACT CGG ATG TGA AG-3’
	R	5’-GAA GGA GTC CTG GCA GTG AG-3’
**TGF-β**	F	5’-TAC GTC AGA CAT TCG GGA AGC A-3’
	R	5’-AGG TAA CGC CAG GAA TTG TTG C-3’
**TNF-α**	F	5’-GCT GAG CTC AAA CCC TGG TA-3’
	R	5’-CCG GAC TCC GCA AAG TCT AA-3’
**IFN-γ**	F	5’-AGA CAA TCA GGC CAT CAG CA-3’
	R	5’-TGC CGT CTC ACC TCA AAC TT-3’
**MCP-1**	F	5’-CCA ATG AGT AGG CTG GAG A-3’
	R	5’-TCT GGA CCC ATT CCT TCT TG-3’
**IL-12**	F	5’-GAT GAC ATG GTG AAG ACG GC-3’
	R	5’-AGG CAC AGG GTC ATC ATC AA-3’
**IL-15**	F	5’-GAT TGA AGG GAA GCA ACG GG-3’
	R	5’-GCA CTC TCC AAC CCA CTT GA-3’
**IL-18**	F	5’-GCC TCA AAC CTT CCA AAT CA-3’
	R	5’-TGG ATC CAT TTC CTC AAA GG-3’

### 2.11 Enzyme-linked immunosorbent assay

Cytokine secretion (TGF-β and IFN-γ) in the tumor tissues was detected using an ELISA kit (BD biosciences Inc., San Diego, CA, USA) according to the manufacturer’s instructions. Proteins were extracted from tumor tissues using RIPA buffer and quantified with a BCA assay.

The results are expressed as pg of cytokine normalized per mg of total protein.

### 2.12 Flow cytometry analysis

Tumors were digested with Collagenase D (1 mg/mL; Sigma-Aldrich, St. Louis, MO, USA) supplemented with DNase1 (1 mg/mL; Sigma-Aldrich) solution in serum-free medium for 15 min at 37°C with a shacking incubator. The tissues were dissociated using a MACS dissociator and MACS C tube (Miltenyi Biotec, Auburn, CA, USA). The tissues were then filtered using a 40-μm cell strainer (Corning Incorporated, Corning, NY, USA) to obtain a single-cell suspension. Erythrocytes were lysed by incubation with 1× red blood cell (RBC) lysis buffer (Invitrogen, Carlsbad, CA, USA) for 5 min at room temperature. Cells were washed and resuspended in staining buffer (BD bioscience, San Jose, CA, USA). After cell count, 1 × 10^6^ cells were stained for 30 min at 4°C with antibodies.

The following antibodies were purchased from BD Biosciences for the identification of monocytes/macrophages: mouse CD45-FITC, F4/80-BV421, CD86-PE-Cy7, and CD206-APC. The M1/M2 ratio was calculated as the percentage of each double positive M1 and M2 macrophages; to identify infiltration of CD 8 T cells, mouse CD45-FITC, CD 8-APC-Cy7, PD1-PE-Cy7, and LAG3-APC; to identify NK cell infiltration, mouse CD45-FITC, CD3e-BV510, NK1.1-PE, IFNγ-PerCP-Cy7, CD107a-APC, and Granzyme B-PE-Cy7.

All antibodies were stained for 30 min in staining buffer. For intracellular staining, the cells were treated with 1× fixation and permeabilization buffer (BD Biosciences) for 30 min. The single-cell suspension was washed and stained with interferon (IFN)-γ and Granzyme B. Flow cytometric analysis was performed using a BD FACSLyric (BD Biosciences).

### 2.13 Statistical analysis

All data are representative of three independent experiments. The data collected were analyzed using Prism 5.01 software (GraphPad Software Inc., San Diego, CA, USA), and are expressed as the mean ± standard deviation (SD). All data were tested for normality using the normality test in GraphPad prism. Unpaired Student’s t-test was used to determine significant differences between two groups. Kaplan-Meier survival analysis was performed in the mouse melanoma model. The statistical significance of the survival between mice treated with PBS and M or DM1 or MDM1 was determined using the log-rank (Mantel-Cox) test. One-way analysis of variance (ANOVA) followed by Tukey’s *post-hoc* test or two-way ANOVA followed by Bonferroni *post-hoc* test was performed for group comparisons. P < 0.05 was considered to indicate a statistically significant difference.

## 3 Results

### 3.1 Conjugation of DM1 to maleimide modified M and characterization of M-DM1

**Figure 1:**
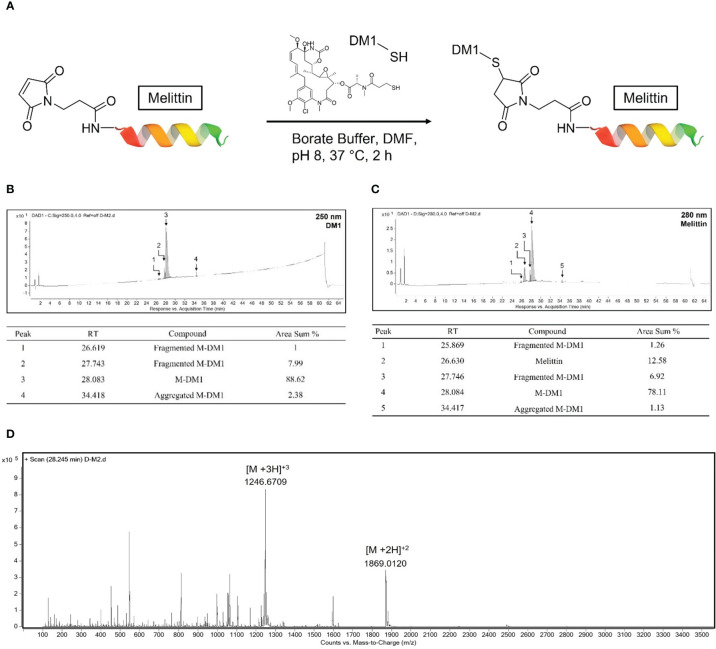
Conjugation of mertansine (DM1) to maleimide modified M and characterization of M-DM1. **(A)** Schematic representation of M-DM1. **(B)** RP-HPLC-DAD chromatogram of product at 250 nm for determination of DM1 and **(C)** at 280 nm for determination of M. **(D)** Charge state distribution of M-DM1 ions (Z = 2, 3).

For the conjugation of the hydrophilic peptide maleimide modified M to the hydrophobic DM1, the reaction was carried out in a buffer containing 10% v/v DMF (**Figure 1A**). DM1 that was not combined with M and DMF was removed using an ultrafilter. The synthesized M-DM1 was analyzed using an RP-HPLC-DAD system; a wavelength of 250 nm represents DM1 (**Figure 1B**), whereas a wavelength of 280 nm represents M (**Figure 1C**). The HPLC-DAD chromatogram showed that DM1 was bound to M, and there were small amounts of fragmented M-DM1, aggregated M-DM1, and unbound M. The mass of M-DM1 was confirmed using a Q-TOF mass spectrometer (**Figure 1D**). The M of [M+3H]+3 and [M+2H]+2 was 3737.0128, which was different from the theoretical mass value by 10 ppm. The concentration used in the biological experiment was calculated using the Beer-Lambert Law using the extinction coefficient of M at 280 nm, and the purity was 78.11% (**Figure 1C**).

### 3.2 M-DM1 induced apoptosis in M2-like TAMs

**Figure 2:**
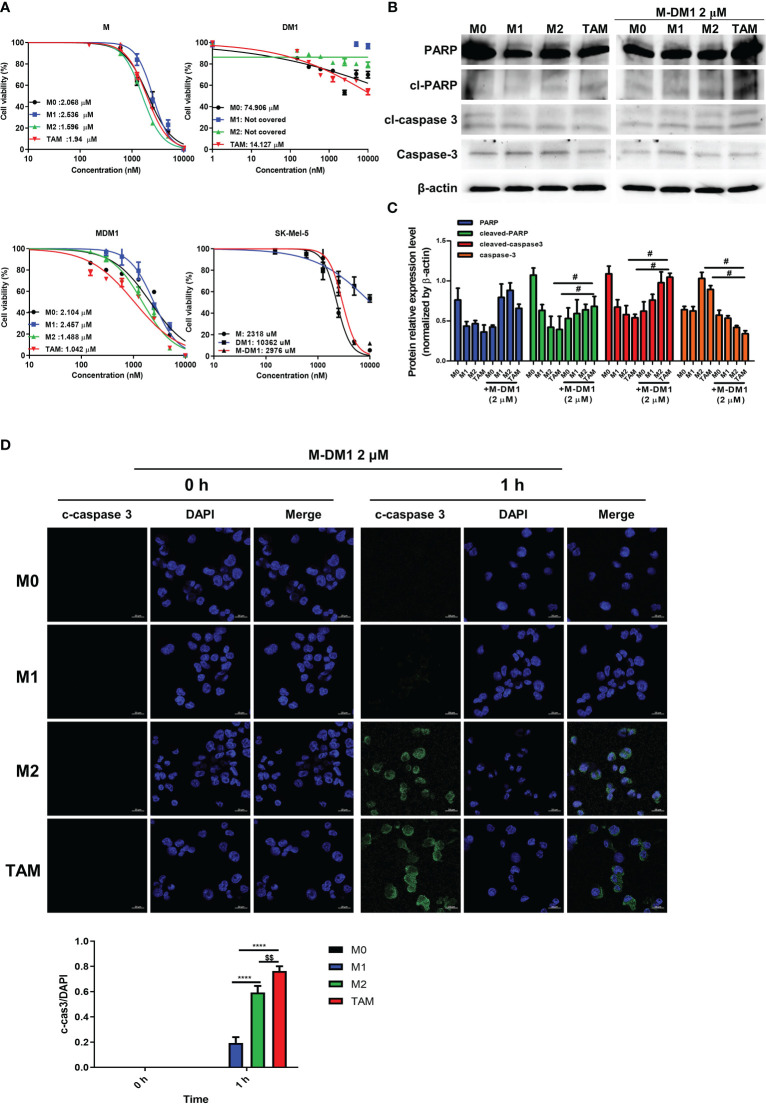
M-DM1 induced apoptosis of M2–like tumor-associated macrophages (TAMs). **(A)** Human monocytes (THP-1) (3 × 10^4^ cells/well) were seeded in a 96-well plate. The cells were treated with 100 nM phorbol-12-myristate-13-acetate for 24 h and polarized into M1, M2 and M2-like TAMs. SK-MEL-5 cells were seeded at a density of 0.5 × 10^4^ cells/well in a 96-well plate. Peptides (10, 5, 2.5, 1.25, 0.612, 0.3, and 0.15 μM) were added into each well along with a serum-free medium and incubated for 24 h Cell viability for macrophages with peptides was assessed using an MTS assay. **(B)** After the treatment of polarized macrophages with M-DM1 for 24 h, proteins were extracted using RIPA buffer. Through western blotting, the expression of apoptosis-related markers PARP, cleaved-PARP, cleaved-caspase-3, and caspase-3 was examined. **(C)** The expression of target proteins was normalized to β-actin expression. The data are presented as the mean ± SD. One-way ANOVA followed by Tukey’s *post-hoc* test was performed for group comparisons; #p < 0.05 vs untreated TAM group. **(D)** Cells were treated with M-DM1 (2 μM) for 1 h and stained for cleaved-caspase-3. The data are presented as the mean ± SD. One-way ANOVA followed by Tukey’s *post-hoc* test was performed for group comparisons; $$p < 0.01 vs M2 group; ****p < 0.0001 vs M1 group.

To evaluate the selective cytotoxicity of M-DM1 against M0, M1, and M2 macrophages and TAMs, the THP-1-derived macrophages were treated with different concentrations of M, DM1, and M-DM1 (0.05–10 μM) after differentiation into M0, M1, M2, and TAMs. A cytotoxicity assay was performed using the MTS assay. The half-maximal inhibitory concentrations (IC_50_) of M-DM1 were 2.104, 2.457, 1.488, and 1.042 μM for M0, M1, M2 macrophages, and TAMs, respectively. M-DM1 induced the apoptosis of M2-like TAMs at low concentrations compared to that observed with M2 macrophages, whereas DM1 alone did not have a cytotoxic effect on any of the macrophages (**Figure 2A**). To test the cytotoxicity of the peptides toward cancer cells, we treated SK-Mel-5 human melanoma cells with the three peptides, and observed that M and M-DM1 were not toxic at concentrations lower than 10 μM, suggesting that M-DM1 was not directly toxic to cancer cells (**Figure 2A**). The expression of PARP, caspase-3, and cleaved caspase-3, which are apoptosis markers, was examined using western blotting (**Figure 2C**). Compared to the untreated TAMs, TAMs treated with M-DM1 showed an increase in the activated form of PARP and cleaved caspase-3. To confirm that apoptosis was induced in M2 macrophages, we stained differentiated macrophages with anti-cleaved caspase-3 antibody after incubation with each drug for 1 h. M-DM1 had little effect on apoptosis induction in M0 and M1 macrophages. In contrast, a considerable increase was observed in the percentage of M2 macrophages stained with cleaved caspase-3 after M-DM1 treatment, and a greater increase was observed in TAMs compared to M2 macrophages (**Figure 2D**). These results demonstrate that M-DM1 specifically induced apoptosis in M2 macrophages and TAMs.

### 3.3 M-DM1 inhibits cell invasion, migration, and proliferation of human melanoma cells by modulating M2-like TAMs

**Figure 3:**
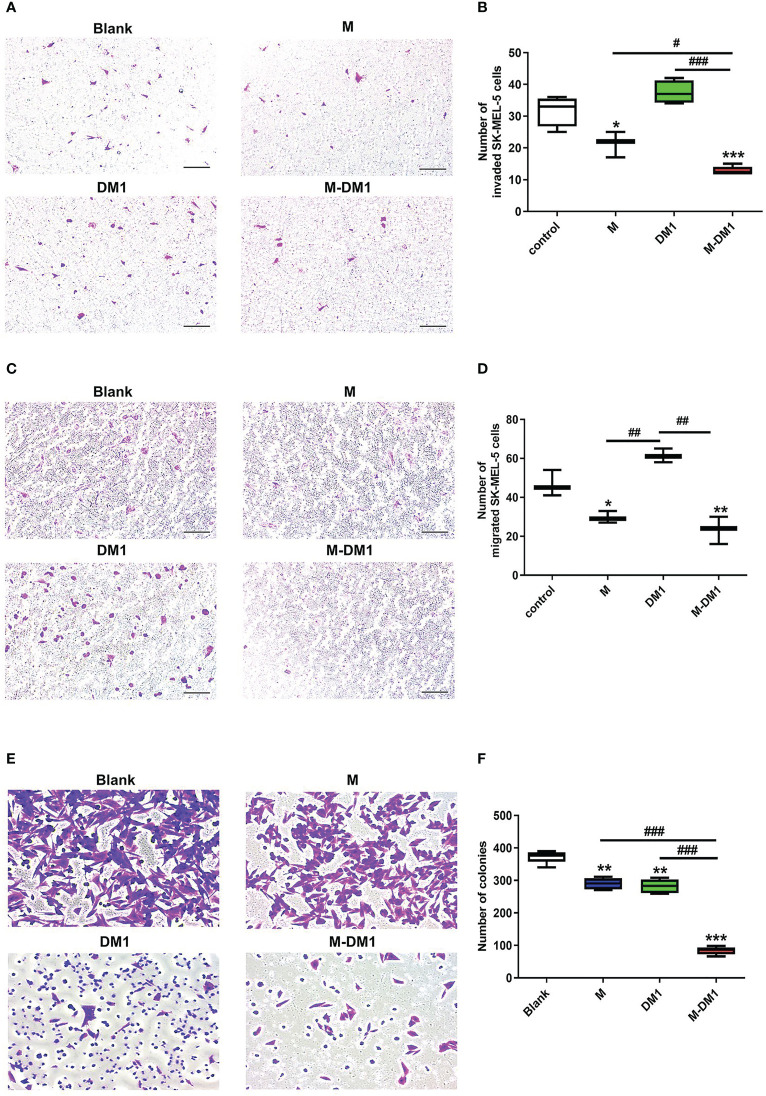
M-DM1 inhibits cell invasion, migration, and proliferation of human melanoma cells by modulating M2-like TAMs. The invasive, migration, and proliferation abilities of SK-Mel-5 cell lines were analyzed using a transwell assay. For invasion assays, Matrigel diluted in serum-free medium (1:9) was added to the upper chamber 24 h prior to initiation. For **(A)** invasion and **(C)** migration assays, SK-MEL-5 cells (5 × 10^4^ cells/well) were seeded in 24 trans-well plates. SK-MEL-5 cells were seeded in the upper chamber, and the TAM supernatant and M-DM1 (2 μM) were added to the bottom chamber. After 24 h of incubation, both chambers were washed with PBS and fixed with 4% paraformaldehyde. These chambers were then stained using 1% crystal violet for 20 min and counted. **(A)** Treatment with M-DM1 significantly suppressed cell invasion, as determined by transwell invasion assay. **(C)** Treatment with Mel-DM1 significantly suppressed cell migration, as determined by transwell migration assay. Quantitative analysis of **(B)** invaded and **(D)** migrated cells. All data are presented as the mean ± SD (*n =* 3). One-way ANOVA followed by Tukey’s *post-hoc* test was performed for group comparisons; * p < 0.05, ** p < 0.01, *** p < 0.001 vs PBS group; #p < 0.05, ## p < 0.01, ### p < 0.001 vs M and DM1 group. **(E)** Cell proliferation was confirmed using a colony formation assay. Human melanoma cells SK-MEL-5 (1 × 10^5^ cells/well) were seeded in 24 transwell plates. After 24 h of incubation, the bottom chamber was washed and fixed with 4% paraformaldehyde for 15 min. After fixation, the cells were stained with 1% crystal violet for 20 min and counted. **(F)** Quantitative analysis of melanoma cells. All images are shown at 10× magnification. The data are presented as the mean ± SD. One-way ANOVA followed by Tukey’s *post-hoc* test was performed for group comparisons; ** p < 0.01, *** p < 0.001 vs PBS group; ### p < 0.001 vs M and DM1 group.

Next, depending on whether M-DM1 induced apoptosis of TAM, such as M2 (**Figure 2**), we examined the effects of M-DM1 on invasion, migration, and proliferation of human melanoma cells. The invasion and migration abilities of melanoma cells were evaluated using a transwell assay. First, THP-1 cells were differentiated into TAMs using the culture supernatant of SK-MEL-5 cells before treat ing with each peptide. The number of invasive SK-MEL-5 cells to the macrophage milieu treated with M-DM1 was reduced compared to treatment with PBS, M, and DM1 (**Figures 3A, B**). In addition, cell migration assays showed that the number of migrating SK-MEL-5 cells was significantly reduced by M-DM1 treatment compared to treatment with PBS, M, and DM-1 (**Figures 3C, D**). In addition, we co-cultured SK-MEL-5 cells with TAM supernatant to determine whether human melanoma cells inhibited M-DM1-induced proliferation using a colony formation assay. M and DM1 treatments resulted in a slight decrease in the number of proliferating cells. In contrast, treatment with M-DM1 significantly inhibited the proliferation of melanoma cells compared to treatment with PBS, M, and DM1 (**Figures 3E, F**). Along with the results shown in **Figure 2**, these findings indicate that treatment with M-DM1 reduced the invasion, migration, and proliferation of melanoma cells by modulating TAMs.

### 3.4 The anti-tumor effects of M-DM1 in a mouse model of melanoma

**Figure 4:**
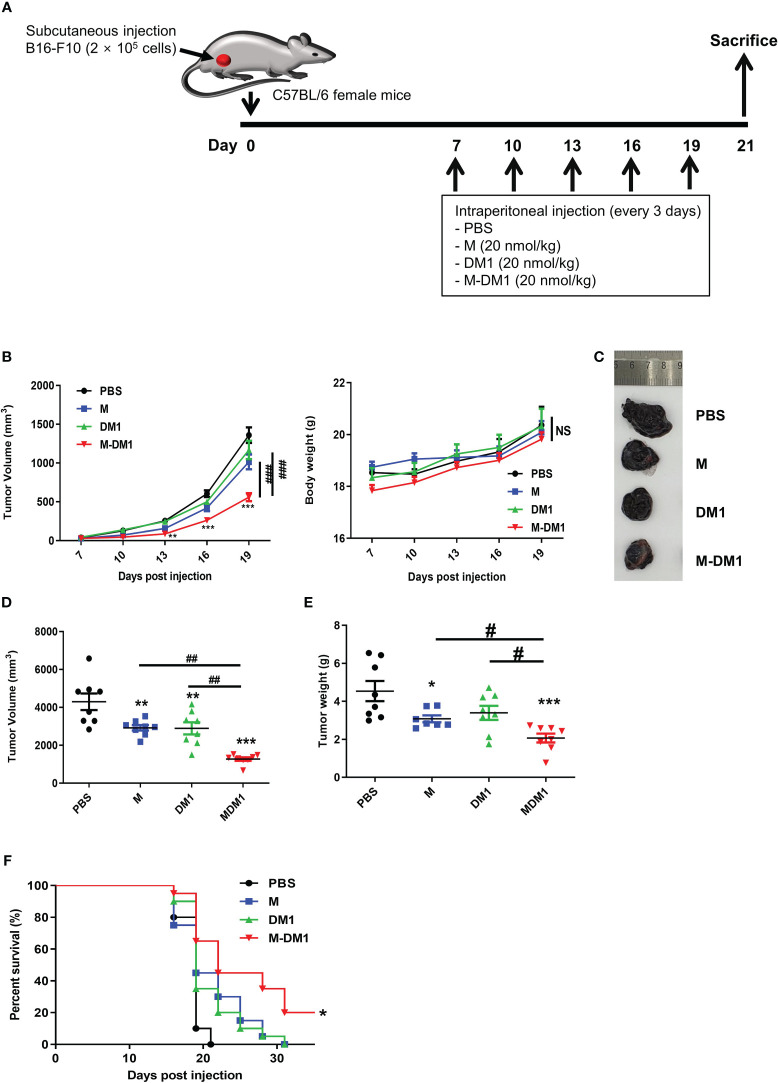
The anti-tumor effects of M-DM1 in a mouse model of melanoma. **(A)** Schematic representation of the experiment. B16F10 mouse melanoma cells were inoculated into the right flank of mice. Seven days after inoculation, each drug was intraperitoneally injected once every 3 days. **(B)** Tumor size and body weight were measured using a digital caliper and an electronic scale, respectively (*n =* 8 mice). The tumor volume was calculated using the following formula: V = (width (2) × length)/2. The data are presented as the mean ± SD. Two-way ANOVA followed by Bonferroni *post-hoc* test was performed for group comparisons; **p < 0.01, ***p < 0.001 vs PBS group; ###p < 0.001 vs M or DM1 group; NS, no significance. To assess tumor volume and weight, the mice were sacrificed on day 21 after tumor inoculation. **(C)** Tumor tissues were imaged, and **(D)** tumor volume and **(E)** weight were measured using a digital caliper and an electronic scale, respectively, after sacrifice (*n =* 8 mice). All data are presented as the mean ± SD. One-way ANOVA followed by Tukey’s *post-hoc* test was performed for group comparisons; *p < 0.05, **p < 0.01, ***p < 0.001 vs PBS group; #p < 0.05, ##p < 0.01 vs M or DM1 group. **(F)** The survival rate following the treatment of the mice with each drug was evaluated by Kaplan-Meier survival analysis in the mouse melanoma model. The median survival and p-values were determined using the log-rank test (Mantel-Cox). The median survival was 19, 19, 19, and 22 in the PBS, M, DM1, and M-DM1 groups, respectively (*n =* 19 mice). *p < 0.05 vs PBS group.

To examine the therapeutic effects of M-DM1 in mice, we used the B16F10 tumor-bearing mouse model. We subcutaneously injected cancer cells into C57BL/6 mice and treated them with 20 nmol/kg M, DM1, or M-DM1 *via* intraperitoneal injections every 3 days (**Figure 4A**). While slight growth inhibition was observed with M and DM1, M-DM1 was found to significantly suppress tumor growth (**Figure 4B**). None of the mice showed any weight loss until the end of the study period, suggesting that there were no significant toxicities due to the drug treatments (**Figure 4B**). At 21 days after tumor cell inoculation, the mice were sacrificed, and tumor volumes and weights were measured. A marked decrease in the tumor volumes and weights was observed in the M-DM1-treated mice group compared to that in the other groups (**Figures 4C–E**). In addition, we determined the survival rate of B16-bearing mice following treatment with each peptide. The median survival of the M-DM1 group was significantly higher than that of the other groups (**Figure 4F**). These results demonstrate that M-DM1 is highly effective in inhibiting tumor growth and prolonging survival in melanoma.

### 3.5 Proliferation of melanoma cells was suppressed by M-DM1 treatment

**Figure 5:**
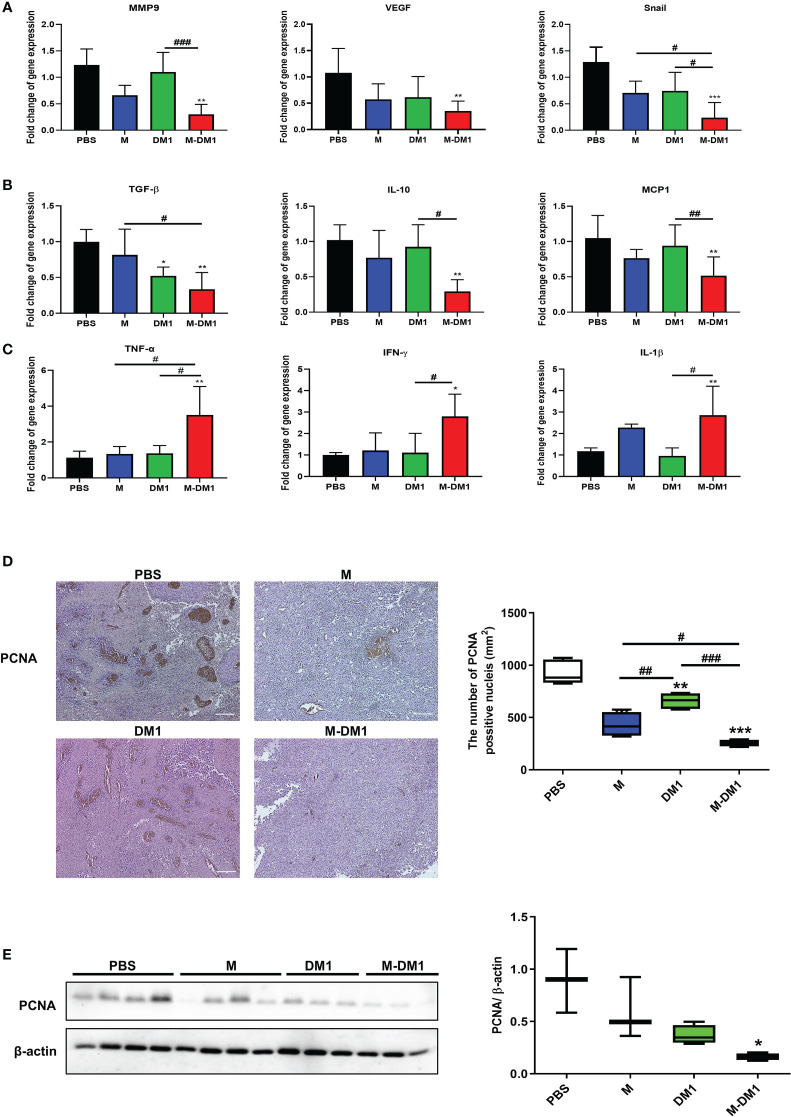
M-DM1 treatment suppresses proliferation of melanoma. **(A)** The expression of epithelial–mesenchymal transition-related markers were analyzed by using qRT-PCR in melanoma tissue (n = 4‒5). **(B)** The expression of anti-inflammatory cytokines markers were analyzed by using qRT-PCR in melanoma tissue (n = 4−5). **(C)** The expression of pro-inflammatory cytokines were analyzed by using qRT-PCR in melanoma tissue (n = 4−5). All data are presented the mean ± SD. One-way ANOVA followed by Tukey’s post-hoc test was performed for group comparisons; *p < 0.05, **p < 0.01, ***p < 0.001 vs PBS group; #p < 0.05, ##p < 0.01, ###p < 0.001 vs M or DM1 group. **(D)** Immunohistochemistry staining was used to analyze the expression of PCNA, a proliferation marker, in tumor tissues (magnification ×10) (n = 4‒5). **(E)** The expression of PCNA in tumor tissues were measured by western blotting (n = 4‒5). All data are presented the mean ± SD. Unpaired Student’s t-test was performed; *p < 0.05, **p < 0.01, ***p < 0.001 vs PBS group; #p < 0.05, ##p < 0.01, ###p < 0.001 vs M or DM1 group.

Since epithelial–mesenchymal transition (EMT) is associated with the migration and invasion of cancer cells, we analyzed the expression of EMT markers, matrix metalloproteinase 9 (MMP9), vascular endothelial growth factor (VEGF), and Snail in tumor specimens using real-time PCR. The mRNA expression levels of *MMP9* and *VEGF* in M-DM1-treated cells decreased markedly (**Figure 5A**). Additionally, as inflammation affects cancer progression in several ways, we investigated whether inflammation-associated cytokines were regulated by M-DM1 treatment at the RNA level. Anti-inflammatory cytokines, such as transforming growth factor (TGF)-β, interleukin (IL)-10, and Monocyte Chemoattractant Protein (MCP)-1, were found to be significantly reduced by M-DM1. On the other hand, M-DM1 markedly upregulated the mRNA expression of proinflammatory cytokines, such as tumor necrosis factor (TNF)-α, interferon (IFN)-γ, IL-1β, and IL-8 (**Figures 5B, C**). Specifically, M-DM1 showed more pronounced growth inhibitory effects than any other group both *in vitro* and *in vivo* (**Figures 3**, **4**). Based on these results, we performed immunohistochemistry to confirm the expression of PCNA as a proliferation marker in tumor tissues. PCNA expression in tissues treated with DM1 did not change compared to that in the control group, whereas M-DM1 showed a decrease in the number of PCNA stained by approximately 70%. M treatment also resulted in a 50% reduction in PCNA expression compared to that in the control group (**Figure 5D**). Similarly, western blotting showed that the expression of PCNA significantly decreased upon M-DM1 treatment (**Figure 5E**). These results suggest that M-DM1 shows a high modulating ability for tumor proliferation compared to M and DM1.

### 3.6 M-DM1 downregulates M2 macrophage infiltration in melanoma tissues

**Figure 6:**
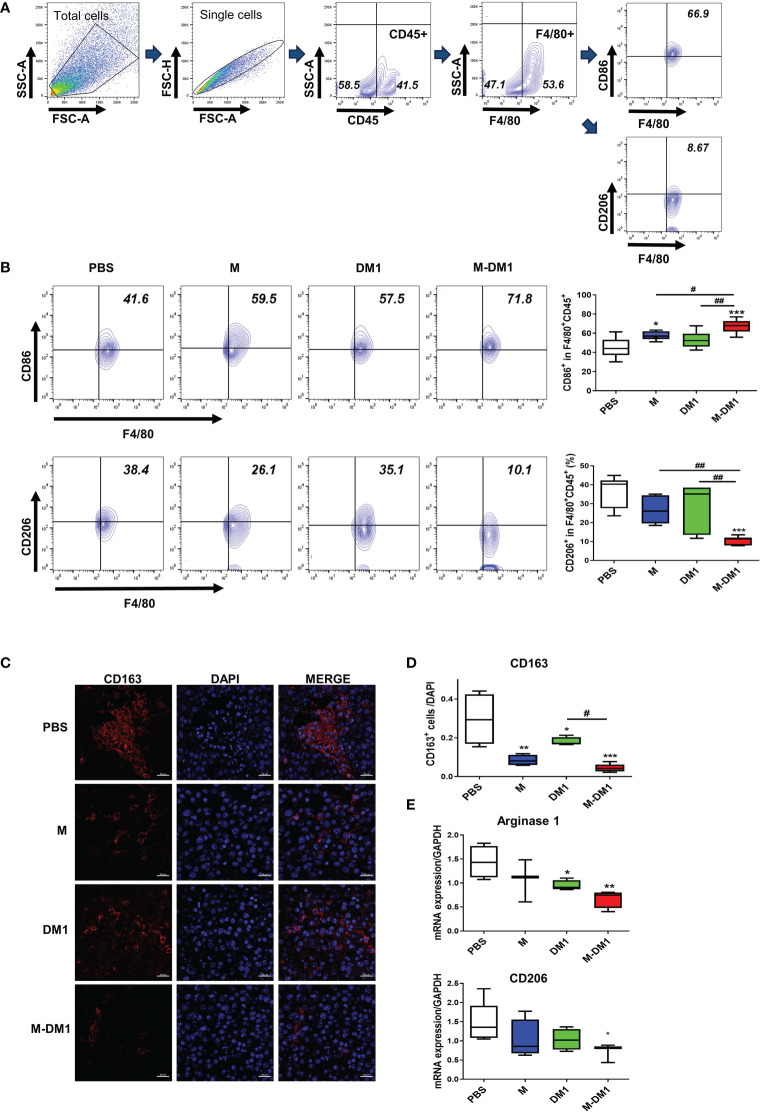
M-DM1 downregulates the population of M2 macrophages in melanoma tissues. After the *in vivo* test, the mice were sacrificed and the tumor tissue was harvested. **(A)** Macrophages from tumor-bearing mice were stained for pan macrophages (F4/80) and CD45^+^ cells. **(B)** The expression of CD86 (M1 macrophage, upper panel) and CD206 (M2 macrophage, bottom panel) was analyzed by flow cytometry (*n =* 7). **(C)** The expression of CD163 in tumor tissues was determined by immunofluorescence staining. Tissue slides were stained for CD163 (red) and nucleus (DAPI; blue) (scale bar *=* 20 μm). **(D)** The graph represents CD163- positive cells relative to the number of nuclei. **(E)** The expression of *arginase 1* and *CD206* mRNA in tumor tissues was analyzed by real-time PCR (*n =* 5). All data are presented as the mean ± SD. One-way ANOVA followed by Tukey’s *post-hoc* test was performed for group comparisons; *p < 0.05, **p < 0.01, ***p < 0.001 vs PBS group; #p< 0.05, ## p < 0.01 vs M or DM1 group.

We previously reported that M binds to M2 macrophages in tumor tissues (28). Based on these findings, we evaluated the effect of M-DM1 on M2 macrophages and tumor growth inhibition. To evaluate changes in the macrophage population in tumor tissues, melanoma cells were isolated from mouse melanoma tissues. Next, the single-cell suspension was stained for M1 (CD86) and M2 (CD206) macrophage markers. The population of CD206^+^F4/80^+^CD45^+^ cells was significantly reduced in the M-DM1 treatment group, although CD86^+^F4/80^+^CD45^+^ cells were slightly increased in all groups compared to that in the PBS group (**Figures 6A, B**). Consequently, the M1/M2 ratio was upregulated. We further confirmed changes in CD163^+^ M2 macrophages in the TME using immunofluorescence analysis (**Figures 6C, D**). In addition, to further confirm reduction in the population of M2 macrophages by M-DM1 treatment, the mRNA expression of M2 markers arginase-1 and CD206 was examined by qRT-PCR, and was found to be significantly downregulated. Both M and DM1 showed a slight decrease in M2 macrophages, although this decrease was not statistically significant (**Figure 6E**).

### 3.7 M-DM1 treatment effectively increased cytotoxicity T cell infiltrations in tumor microenvironments

**Figure 7:**
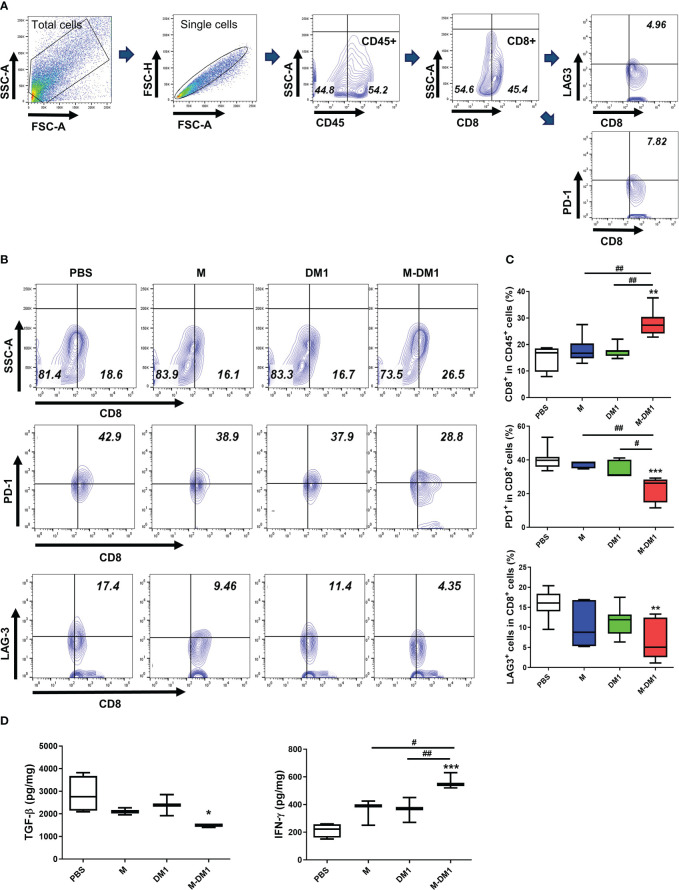
M-DM1 treatment effectively regulates the activity of infiltrated cytotoxic T cells and downregulates T cell exhaustion. Cells were isolated from tumor tissues. **(A)** The gating strategies of T cells. **(B)** CD8+ T cells were stained with CD45 and CD8 (upper panel), and T cell exhaustion markers, PD-1 and LAG-3, as CD45^+^ CD8^+^ PD-1^+^ or CD45^+^ CD8^+^ LAG-3^+^ (middle and bottom panels, respectively). Cells are shown as contour within the CD8^+^ and PD-1^+^ or LAG-3^+^ axis gated on CD45^+^ cells (*n =* 7). **(C)** Data are displayed as the percentages of CD8^+^ (upper), PD-1^+^ CD8^+^ (middle) and LAG-3^+^ CD8^+^ cells (bottom) in the CD45^+^ cell population. **(D)** Cytokine (TGF-β and IFN-γ) analysis was confirmed by ELISA. Proteins were harvested from tumor tissues by RIPA buffer. All data are presented as the mean ± SD. One-way ANOVA followed by Tukey’s *post-hoc* test was performed for group comparisons; *p < 0.05, **p <0.01, ***p<0.001 vs PBS group; #p < 0.05, ##p < 0.01 vs M or DM1 group.

Infiltrated CD8 T cells in the TME play a crucial role in regulating tumor growth (36, 37). The exhaustion and functional impairment of CD8 T cells in the TME is a key feature of various cancers. To determine whether M-DM1 treatment elicited any effect on T cell exhaustion, PD-1- and LAG3-positive CD8 cell populations in the TME were assessed by flow cytometry. In the CD8 ^+^ T cell population, M-DM1 treatment resulted in decreased expression of both PD-1 and LAG3. Notably, the total population of CD8^+^ T cells was markedly elevated following M-DM1 treatment (**Figures 7A–C**), while M or DM1 treatment alone did not affect CD8^+^ T cell infiltration in the TME. Using ELISA, we measured the levels of TGF-β and IFN-γ. The decrease in TGF-β production after M-DM1 treatment was significant. In contrast, the production of IFN-γ was upregulated following M-DM1 treatment compared to that in the control group (**Figure 7D**). These results suggest that M-DM1 elevates the infiltration of CD8^+^ T cells in the TME and increases the secretion of CTL-responsive cytokines, thereby inhibiting tumor progression.

### 3.8 M-DM1 regulates the activation of infiltrated NK cells by regulating the secretion of related cytokines

**Figure 8:**
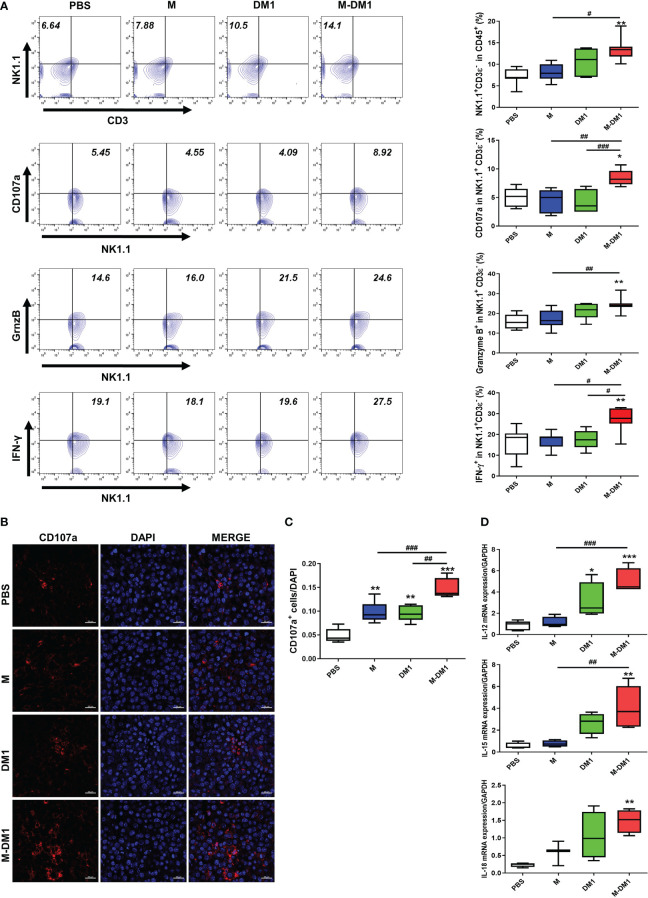
M-DM1 regulates the activation of infiltrated NK cells by regulating the secretion of related cytokines. **(A)** Expression of NK 1.1, CD107a, Granzyme B, and IFN-γ in CD45- positive cells upon treatment with M, DM1, and M-DM1. Representative contours are shown (*n = 7*). **(B, C)** The expression of CD107a as a marker of NK cell functional activity was evaluated through immunofluorescence staining (scale bar = 20 μm). **(D)** Cytokines, such as IL-12, IL-15, and IL-18, related to NK cell survival and proliferation, were examined by real-time PCR (*n =* 5). All data are presented as the mean ± SD. One-way ANOVA followed by Tukey’s *post-hoc* test was performed for group comparisons; *p < 0.05, **p < 0.01, ***p < 0.001 vs PBS group; #p < 0.05, ##p < 0.01, ###p < 0.001 vs M or DM1 group.

Given the upregulation of IFN-γ expression observed at both the mRNA and protein levels (**Figure 7**), we investigated whether an increase in IFN-γ production by M-DM1 affects the induction of infiltrated NK cells in melanoma tissues. We stained and detected NK1.1-, CD107a-, Granzyme B-, and IFN-γ- positive cells in the CD3ε- negative population by flow cytometry. A notable increase was observed in the number of NK 1.1^+^ cells in the M-DM1 group, whereas a slight increase was observed with both M and DM1. The populations of CD107a-, Granzyme B-, and IFN-γ-positive NK cells increased following M-DM1 treatment (**Figure 8A**). In addition, we evaluated CD107a expression in the tumor tissues using immunofluorescence staining. Similarly, CD107a staining in M-DM1-treated melanoma tissues was significantly increased compared to that in the other groups (**Figures 8B, C**). Next, the expression of IL-12, IL-15, and IL-18, which are pro-inflammatory and immune regulatory cytokines, was measured at the mRNA level. IL-12, IL-15, and IL-18 levels were found to be significantly increased in the M-DM1 treatment group compared to the PBS treatment group. The expression of each cytokine showed a tendency to increase in the order of M, DM1, and MDM1 treatment (**Figure 8D**). Taken together, these data indicate that M-DM1 modulatesthe infiltration of NK cells, which play a crucial role in inhibiting tumor growth in a mouse model of melanoma.

## 4 Discussion

TAMs in the TME have been classified into M1-like and M2- like phenotypes (38, 39). In particular, M2-like TAMs secrete immunosuppressive factors to inhibit the cytotoxic activity of immune cells with anti-tumor properties, such as cytotoxic T cells and NK cells (40–42). In melanoma, the density of M2-like TAMs is correlated with the invasiveness of tumor cells and a poor prognosis. According to Huang et al. (43) and Fridlender et al. (44), a reduction in TAMs inhibits the growth of control tumors. Therefore, the depletion or reduction of M2-TAMs is a therapeutic strategy for the inhibition of tumor progression (45–47).

M, a natural compound in bee venom, has a wide range of biological activities and may have applications in modern medicine (48). Recently, many studies have been conducted on the effects of M on the induction of apoptosis in various cancers, such as breast, ovarian, prostate, and lung cancer (43, 49). Previously, we reported that M preferentially acts on M2 macrophages, which inhibit tumor growth by targeting M2-like TAMs in lung models (28). Although the mechanism by which M selectively acts on M2 macrophages has not been elucidated, it may suggest a role for carrier peptides as a novel therapeutic strategy for targeting M2-like TAMs.

DM1 inhibits the polymerization of tubulin and can be used for the treatment of diverse carcinoma, including lung cancer, multiple myeloma, melanoma, and breast cancer. Therefore, we synthesized M as a vehicle for M2-like TAMs and DM1 as a payload to enhance the therapeutic effects as a PDC on melanoma cells. In the present study, M-DM1 exhibited cytotoxic activity and induced the activation of cleaved caspase-3 and cleaved PARP, which are known apoptosis markers, selectively in M2 macrophages as opposed to M0 and M1 macrophages. These results suggest that M-DM1 selectively targets M2 macrophages. To support these results, further research should be conducted on the mechanisms by which M-DM1 preferentially targets M2-like TAMs.

Based on our *in vitro* results, we compared the effects of M, DM1, and M-DM1 *in vivo*. A superior effect on the inhibition of tumor growth was observed in M-DM1 compared to M and DM1. Furthermore, the survival rate was found to increase in the M-DM1 group compared to the M and DM1 groups. Our data suggest that the novel drug M-DM1, which conjugates M and DM1, exhibits improved anticancer function. Currently, no toxicity test for the synthesized drug has yet been conducted. As a follow-up experiment, the safety of the new drug should be evaluated, including toxicity testing of drugs in mice.

Melanoma is the most aggressive skin tumor; it is derived from melanocytes in the skin and is prone to metastasis. It has a poor overall survival, and various attempts have been made to solve this issue (50, 51). Moreover, recent studies have reported that M2 macrophages are associated with the aggressive behavior of melanoma (52, 53). Therefore, to develop new treatments for melanoma, targeting M2 macrophages is necessary to influence the key mechanistic events that regulate melanoma, including cell proliferation, survival, invasion, and metastasis. In the present study, we demonstrated the inhibition of proliferation, migration, and invasion of melanoma cells by conditioned medium containing M-DM1. Additionally, we confirmed that the expression of EMT markers, such as Snail, MMP9, and VEGF, was significantly reduced *in vivo* by M-DM1 treatment. PCNA, a marker of cell proliferation, was also decreased in the M-DM1 group compared to that in the PBS group. Furthermore, CD206^+^ and CD163^+^ cells, which are markers of M2 macrophages, were significantly suppressed in the M-DM1 group in tumor tissues. These results indicate that M-DM1 can efficiently inhibit the proliferation and EMT by targeting M2-like TAMs in melanoma.

Numerous studies have reported that a subset of TAMs suppresses the anti-tumor responses of tumor-infiltrating lymphocytes and is associated with poor outcomes in patients with cancer (54, 55). Dysfunctional CD8^+^ T cells in cancer are characterized by high expression of inhibitory receptors, including PD-1, TIM-3, and LAG-3, which are positively associated with T cell exhaustion. T cell dysfunction results in impaired proliferation and a decreased production of effector molecules in response to tumor antigens. Blocking these molecules could be considered a novel strategy to further enhance the efficacy of immunotherapy by restoring T cell function in tumors (56, 57). Therefore, it is important to understand how CD8^+^ T cell functions are modulated for future therapeutic development and T cell-mediated immunotherapy. In the present study, PD-1^+^ LAG-3^+^ CD8^+^ cells were downregulated; in contrast, CD8^+^ cell infiltration in the TME was upregulated by M-DM1 treatment. Accordingly, our results illustrated that reduced M2-like TAMs successfully mediated “non-exhausted” CD8^+^ T cell infiltration within tumors. Infiltrated T cells can efficiently enhance the immune response, and thus cytotoxic activity, against tumor cells.

In the TME, infiltrated NK cells are known to inhibit tumor growth. Intratumoral infiltrated NK cells have been reported to be directly associated with increased survival (58, 59). Therefore, strategies to upregulate the infiltration of NK cells into tumors may enhance their anti-tumor efficacy. Our data showed that M-DM1 induced the upregulation of NK1.1^+^ cells, CD107a^+^ cells, IFN-γ^+^ cells, and Granzyme B^+^ cells in the CD3^-^ T cell population. These results strongly suggest that M-DM1-mediated tumor suppression could be attributed to increased CD8^+^ T cell and NK cell infiltration in the TME. However, further study is needed to elucidate the function of effector cells in the anti-tumor effect of M-DM1 in melanoma. Our data also showed that M-DM1 treatment induced the production of IL-12, IL-15, and IL-18, which are cytokines known to support NK cell maturation, and increased infiltration of NK cells in response to the treatment. We showed that NK cell infiltration in the TME was more efficiently controlled by M-DM1 treatment. We observed that M-DM1 modulates the functions of effector cells, such as cytotoxic T cells and NK cells, in the TME, suggesting that M-DM1 could have potent anti-tumor efficacy compared to M and DM1.

## 5 Conclusions

In summary, we constructed a novel PDC, M-DM1, by combining M and DM1. Our study shows that M-DM1 can inhibit tumor progression, invasion, and migration by alleviating M2-like TAMs in the melanoma TME. We also demonstrated that M-DM1 could prolong the survival rate of melanoma compared to M and DM1 alone. M-DM1 can regulate not only M2 macrophages but also the infiltration of other effector cells, such as CD8^+^ T cells and NK cells, to enhance anti-tumor effects. Hence, M-DM1 is a promising anti-tumor agent that suppresses M2-like TAMs in the TME.

## Data availability statement

The original contributions presented in the study are included in the article/supplementary materials. Further inquiries can be directed to the corresponding authors.

## Ethics statement

The animal study was reviewed and approved by Institutional Animal Care and Use Committee of Kyung Hee University.

## Author contributions

CJ, I-HH, and HB designed this study. CJ, SK, HK, and IC analyzed and interpreted the data. CJ and I-HH wrote the original draft of the manuscript. JK synthesized compound M-DM1. I-HH provided technical support for the experiments. CJ, I-HH, J-HJ, and HB revised the manuscript. J-HJ and HB supervised the study. All authors contributed to the article and approved the submitted version.
